# Contrastive learning and advanced bundle metrics outperform fractional anisotropy for Alzheimer’s disease diffusion MRI prediction: findings from the Beyond FA challenge

**DOI:** 10.1117/1.JMI.13.5.054003

**Published:** 2026-10-15

**Authors:** Elyssa McMaster, Nancy Newlin, Trent Schwartz, Adam Saunders, Gaurav Rudravaram, Jiheng Li, André Hucke, Yehyun Suh, Jongyeon Yoon, Eleanor Liard, Michael Kim, Chenyu Gao, Karthik Ramadass, Chloe Cho, Sriram Gnana Sambanthan, Tomasz Pieciak, Dominika Ciupek, Santiago Aja-Fernández, Antonio Tristán-Vega, Maciej Malawski, Alex Caron, Antoine Théberg, Francois Reault, Arnaud Boré, Manon Edde, Ji Kim, Yihao Liu, Lianrui Zuo, Eleftherios Garyfallidis, Talia Nir, Neda Jahanshad, Kurt Schilling, Daniel Moyer, Bennett A. Landman

**Affiliations:** a Vanderbilt University, Department of Electrical and Computer Engineering, Nashville, Tennessee, United States; b Vanderbilt University, Department of Computer Science, Nashville, Tennessee, United States; c Université Paris-Panthéon-Assas; Paris, France; d Vanderbilt University, Department of Biomedical Engineering, Nashville, Tennessee, United States; e Indian Institute of Technology Madras, Chennai, India; f Universidad de Valladolid, Valladolid, Spain; g Sano Centre for Computational Medicine, Kraków, Poland; h Université de Sherbrooke; Sherbrooke Connectivity Imaging Laboratory (SCIL), Informatics Department, Science Faculty, Sherbrooke, Quebec, Canada; i Université de Sherbrooke; Videos & Images Theory and Analytics Laboratory, Faculty of Science, Sherbrooke, Quebec, Canada; j McGill University, Douglas Mental Health University Institute, StoP-AD Centre, Montreal, Quebec, Canada; k Creighton University School of Medicine, Omaha, Nebraska, United States; l Indiana University, Luddy School of Informatics, Computing, and Engineering, Bloomington, Indiana, United States; m University of Southern California, Mark and Mary Stevens Neuroimaging and Informatics Institute, Keck School of Medicine, Los Angeles, California, United States; n Vanderbilt University Institute of Imaging Science, Vanderbilt University Medical Center, Nashville, Tennessee, United States; o Vanderbilt University Medical Center, Department of Radiology and Radiological Sciences, Nashville, Tennessee, United States

**Keywords:** diffusion MRI, harmonization, microstructure, connectome, feature extraction

## Abstract

**Purpose:**

The inclusion of diffusion-weighted magnetic resonance imaging (DW-MRI) in major national studies has allowed for quantification and characterization of white matter macrostructure and microstructure in cases of disease and disorder. Diffusion tensor imaging (DTI) yields 3D scalar maps to summarize the DW-MRI signal with quantitative metrics. Fractional anisotropy (FA) is a DTI metric that describes the degree of anisotropy within diffusion and is frequently used to interpret white matter integrity, but it exhibits high sensitivity in pathological interpretation. These properties of FA make it difficult to use as a consistent candidate imaging biomarker, as it makes different ages, cognitive statuses, and pathologies difficult to discern. With the development of other white matter models and metrics, such as structural connectivity network measures and tractography bundle analyses, there are many combinations to analyze for white matter integrity analysis, especially in the context of lower-quality clinical data that FA’s voxel-wise computation can be sensitive to. The widespread collection of DW-MRI data necessitates an analysis of these metrics to interpret white matter integrity beyond what FA can capture.

**Approach:**

We designed a challenge for the medical imaging community to compare crowdsourced DW-MRI candidate imaging biomarkers to a baseline relying on FA in an age, sex, and cognitive status classification task and variables unknown to teams during the challenge. After the challenge submission period ended, we evaluated methods based on sensitivity to environmental variables (humidity and temperature on the day of the scan), and we correlated features with known Alzheimer’s disease (AD) pathology (amyloid beta 42, tau, and ptau cerebrospinal fluid/CSF readings) to evaluate if the methods collected in this study are robust to the limitations of FA. The key innovations are that we crowdsource DW-MRI feature representations for AD, we develop a framework to compare imaging feature representations in this context, we evaluate the sensitivity of DW-MRI imaging candidate biomarkers to environmental variables, and we correlate the novel features to known AD pathological measures.

**Results:**

We find that bundle-based methods outperform whole-brain methods in age classification tasks, but deep learning, computer vision-based methods show a new avenue for cognitive status prediction tasks.

**Conclusion:**

The submissions to our challenge show that there are many approaches to consider when developing new AD feature representations.

## 1 Introduction

Diffusion-weighted magnetic resonance imaging (DW-MRI) provides a non-invasive modality to image white matter microstructure *in vivo*.^1^ During a DW-MRI scan, magnetic gradients (b-values) are applied in specified spatial directions (b-vectors) to sensitize the random thermal motion of water molecules within tissue to reveal the inherently anisotropic white matter microstructure, or tube-like connective tissue within the brain.^1^ Many international-scale research studies employ DW-MRI as part of their imaging protocol to understand different diseases and disorders that change the brain’s,^2^^–^^9^ which invites an exciting opportunity to model and quantify the white matter. Diffusion tensor imaging (DTI)^10^ offers voxel-wise, tensor-based quantitative measures with known correlations with pathology.^11^^,^^12^ However, due to the heterogeneous nature of both large-scale study scanner hardware and pathological biology, white matter models including DTI often suffer from high variability across sites and cohorts.^13^^–^^15^ Harmonization, or efforts to maximize biologically relevant signal while minimizing site-related variance, across diffusion images and white matter models has shown promise in structural pathological data,^16^ but remains a challenge for multi-site diffusion studies due to protocol differences and scanner hardware limitations.^14^^,^^17^

Despite multisite challenges with DTI, fractional anisotropy (FA) is a tensor-derived metric^18^ widely used in Alzheimer’s disease (AD) characterization.^12^^,^^19^^,^^20^ FA is computed from the three eigenvalues of the diffusion tensor as shown in Eq. (1)^1^

$$\mathrm{FA}=\frac{1}{2}\sqrt{\frac{(\lambda_{1}-\;\lambda_{2}{)}^{2}+(\lambda_{2}-\;\lambda_{3}{)}^{2\;}+(\lambda_{3}-\;\lambda_{1}{)}^{2}}{\lambda_{1}^{2}+\;\lambda_{2}^{2}+\;\lambda_{3}^{2}}},$$ (1)

where $\lambda_{1}$, $\lambda_{2}$, and $\lambda_{3}$ denote the three eigenvalues of the diffusion tensor. When this computation is performed across all voxels, the result is a scalar spatial map in which each voxel has a value between 0 and 1, where 0 represents isotropic diffusion, and 1 represents highly anisotropic diffusion. When this computation is performed across all voxels, the result is a scalar spatial map where every voxel maps to a value between 0 and 1, where 0 represents isotropic diffusion and 1 represents diffusion restricted across one axis.^1^

Although FA may be more clinically feasible than other approaches since DTI requires only six noncollinear diffusion-weighted acquisitions for tensor computations, the tensors are computed on a voxel-wise basis that compounds partial volume effects in images with low or anisotropic spatial resolution.^21^^,^^22^ As a result of limitations from the tensor model, FA is highly sensitive to multiple microstructural features and fiber configurations, limiting its specificity as a feature representation on its own.^20^ With these limitations, we and others have identified the need to consider alternatives to FA in a clinical context,^20^^,^^23^ especially in studies with multiple spatial resolutions included in their protocol or that use historical datasets. Due to limitations between data availability and scanner hardware variability, we have yet to maximize the potential of large-scale white matter model findings related to AD.

Therefore, the future of the study of DW-MRI images requires a comprehensive understanding of white matter models and their sensitivity and specificity to different conditions such as aging and pathology, as well as environmental factors, which are an understudied but relevant potential confounder within large-scale brain imaging.^24^ Specifically, there is a need to identify correlations between metrics and biological and environmental phenomena to develop an interpretation of diffusion data for important aging and pathology studies. The hypothesis is that these alternative metrics beyond FA may improve the interpretation of diffusion data for major use cases such as aging and pathology with higher accuracy. These white matter models may include, but are not limited to, other DTI metrics such as mean diffusivity (MD) and radial diffusivity (RD),^1^ fiber orientation distributions (FODs),^25^ white matter segmentation measurements, tractography bundle metrics,^26^ and complex graph measure representations derived from a whole-brain structural (Fig. 1).^27^^,^^28^ The challenge was designed to encourage participation from researchers in biomedical imaging, neuroscience, and machine learning.

**Fig. 1:**
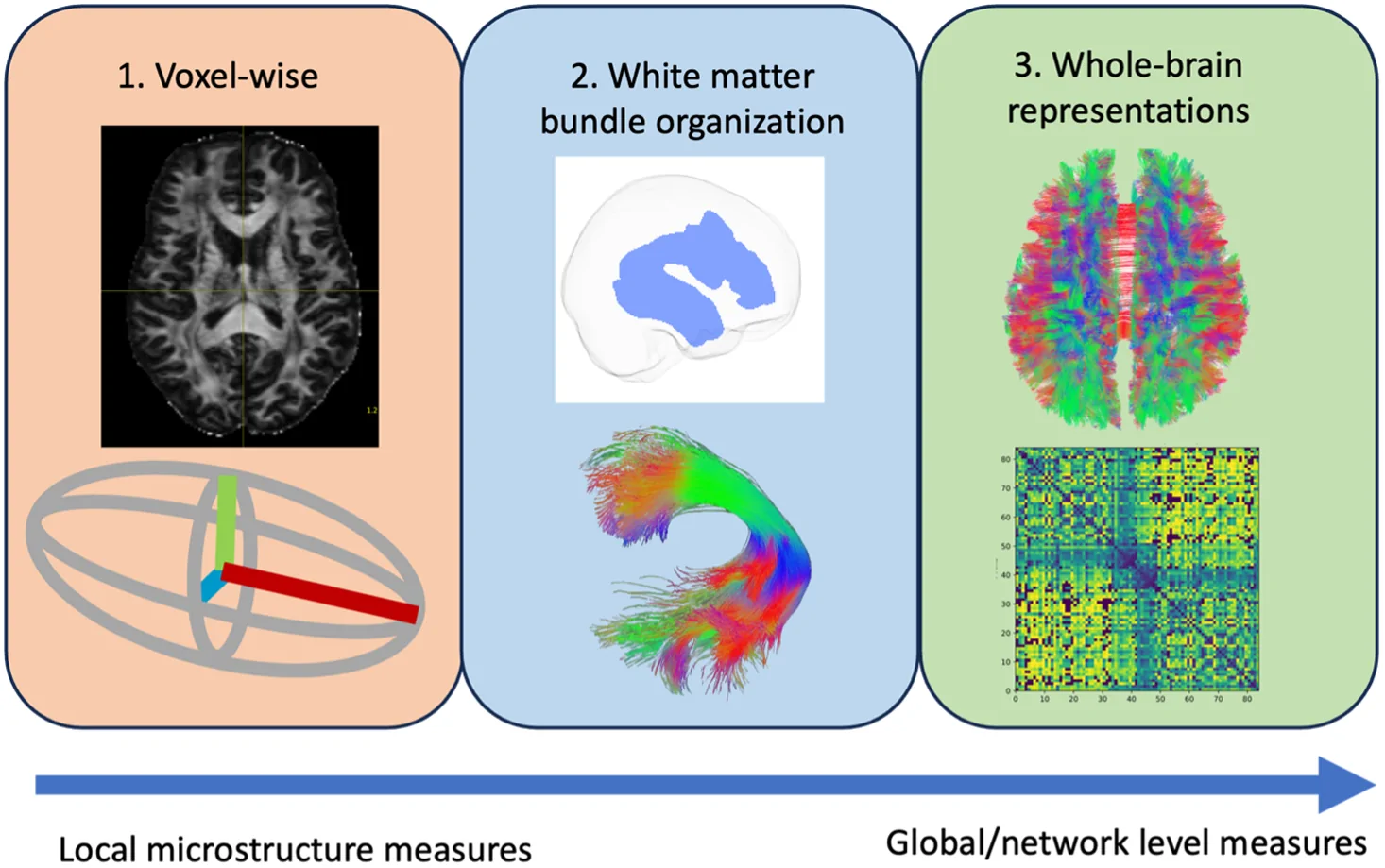
Illustration of possible interpretations for AD-related DW-MRI feature representations.

Alhough much of the literature emphasizes FA and local microstructural measures, our challenge encouraged teams to consider additional representations of the brain, such as bundle-wise brain pathway organization^29^ and whole-brain network measures, to address the well-documented shortcomings of FA as an AD feature representation.

In this challenge, participants could use any diffusion data that pertains to AD and were asked to develop their own method to extract machine learning features that correlate with age, sex, and cognitive status in healthy control and disease cases, but we ask that they limit their training to use a single shell of ∼b≤1000. We proposed a tri-classification between subjects who are cognitively normal (CN), have mild cognitive impairment (MCI), and have AD. This way, we sought to see which metrics show high sensitivity and high specificity, even in clinically feasible data. We did not tell participants that we would also attempt to extract information such as humidity and temperature on the day of the scan and the known AD biomarkers of amyloid beta 42, tau, and ptau.^19^

This challenge takes inspiration from previous DW-MRI and Alzheimer’s-related challenges presented to the medical imaging community. The TADPOLE Challenge (2017 to 2020) invited submissions of any AD-related biomarkers from the ADNI dataset.^8^^,^^30^^,^^31^ TADPOLE attracted a variety of feature selection methods (manual, semi-automatic, or automatic), each of which produced a unique number of features and used unique diagnosis prediction models.^30^ Four of the top five submissions in their “clinical prediction” category used machine learning methods, highlighting the potential of machine learning and deep learning for classification of cognitive status. In 2021, AI4AD^32^ provided participants with a predetermined set of white matter pathways and diffusion measurements and asked for classification methods for cognitive status after identifying a gap of white matter biomarkers within TADPOLE^30^^,^^31^ and reported high accuracy in binary classification tasks with weaker performance in tri-classification (CN, MCI, and AD), which introduced the need to identify more robust biomarkers to differentiate between cognitive statuses^30^^,^^31^ and age-related anomaly detection.^33^^,^^34^ AI4AD addressed a major shortcoming in the field: Although many approaches report high accuracy for image-only classification methods for AD, many report comparisons only for binary classification (CN/MCI, CN/AD, and MCI/AD), which limits addressable learnable representations across cognitive statuses.^12^^,^^34^^,^^35^ Assessing diffusion MRI-derived features on the more difficult prediction task could provide greater insight into the progression of AD as we investigate across the full spectrum of cognitive status. For the QuantConn Challenge at MICCAI 2023,^14^ participants submitted algorithms to harmonize the white matter models of the same subjects scanned with one DW-MRI protocol with high angular resolution and low spatial anisotropy and another protocol with low angular resolution and high spatial anisotropy. Evaluations focused on the minimization of scan-rescan variability based on the submitted algorithm within a nonpathological population, inviting attempts toward robust white matter metrics among diverse populations based on their findings.

In this work, we ask the community to identify robust DW-MRI feature representations in the form of feature vectors, and we compare the extracted features in a common deep learning framework. The key innovations are (1) we evaluate diffusion metrics from both the microstructure and the macrostructure in the context of crowdsourced DW-MRI feature representation development for empirical study, (2) we evaluate the potential for diffusion metrics specific to healthy aging and AD pathology, and (3) we integrate environmental variability as a metric of interest. Our challenge sought the submission of features extracted from diffusion data to correlate diffusion-derived metrics and feature representations.^36^

### 1.1 Challenge

Participants were asked to submit an algorithm that performs single-shell white matter model computations of their choice and then extract 128 meaningful features from each image to correlate with age, sex, and cognitive status. The limit of 128 latent features was chosen as a balance between expressiveness and practicality. It provides sufficient capacity for participants to encode rich representations while preventing excessively large feature vectors that could provide an unfair advantage or create unnecessary computational burdens. A fixed-size representation also ensured that all submissions could be evaluated consistently within the challenge infrastructure. We evaluated the preservation of age and because these variables represent relevant biological characteristics and allowed us to measure whether representations remained sensitive to meaningful AD-related information while other biological characteristics are preserved. We limited data to single-shell suits existing AD DW-MRI scanning protocols.^8^ The cognitive statuses included are cognitively normal/cognitively unimpaired (CN), mild cognitive impairment (MCI), and AD. We adopted a three-class classification task because distinguishing cognitively normal controls, mild cognitive impairment, and AD remains an unresolved challenge in the medical imaging community, as shown by AI4AD. Robust solutions to classify CN, MCI, and AD remain an active area of research. As a separate evaluation, we computed the correlation for other, “secret variables” (environmental variables) that we did not reveal until the end of the challenge. Participants submitted their methods containerized as Docker images^37^ to grand-challenge.org, a challenge hosting site specific to biomedical imaging, to test input/output structure, with final evaluations performed locally. We opened two phases for participants: Phase 1, which is a trial image dataset so that participants can make sure that their formats are compatible with the challenge format. We limited participants to five submissions and two single-shell images from the open MASiVar dataset^38^ available on OpenNeuro that participants can access to debug their code. Phase 2 includes the true evaluation of Docker with a multilayer perceptron (MLP) framework provided to participants to run their own experiments, with a single upload submission chance. We used an MLP as the downstream evaluation model because it is sufficiently expressive to capture nonlinear relationships while remaining lightweight and standardized across all submissions. Using the same evaluation model for every submitted Docker ensured that differences in performance primarily reflected the quality of the learned representations rather than differences in downstream model complexity.

## 2 Data

Due to the sensitive nature of AD data and patient information, we did not provide any data to participants and instead encouraged to request their own access to data and use what they have access to. We intentionally withheld the identity of the source dataset from participants to encourage the development of methods that generalize across AD datasets rather than exploiting dataset-specific characteristics. In addition, the underlying datasets are governed by their respective data use agreements, and we do not have the authority to disclose information beyond what is permitted by those agreements. We suggested that participants request access to such studies as OASIS3,^3^ the Alzheimer’s Disease Neuroimaging Initiative (ADNI),^8^ the Baltimore Longitudinal Study of Aging (BLSA),^5^ the Australian Imaging, Biomarkers, and Lifestyle study of aging (AIBL),^6^ or any in-house dataset available to them. Participants were not asked to share their data with the challenge at any time. Since we aimed to use these approaches to analyze large-scale studies, participants expected that input data would be heterogeneous: different sites, scanners, diffusion protocols (with at least one b0 and with at least six different directions, b-value equal to or less than $1000\;s/{\mathrm{mm}}^{2}$), sexes, and cognitive statuses would be represented in the dataset. Our evaluation included training and testing a shallow multilayer perceptron (MLP) outlined in Sec. 3, “Testing Framework.”

All subjects used to validate the submitted methods come from ADNI^7^ Click or tap here to enter text.. For training, we selected a cross section of 210 CN subjects, 210 subjects with MCI, and 80 subjects with AD. The train/test split was designed to reflect the class imbalance commonly encountered in real-world AD studies, where healthy controls substantially outnumber patients with AD. This evaluation setting emphasizes generalization under realistic conditions and rewards representations that remain effective despite limited disease-specific training data. A successful challenge algorithm should extract meaningful features that highly correlate with age, sex, and cognitive status.

We collected the date and institution of each ADNI scan from within their individual DICOM files and matched each institution with a city and GPS coordinates. We used Open-Meteo^39^^–^^41^ to collect weather information (temperature and humidity) for the day of each scan and, because we do not have the exact time of scan, we averaged the day’s temperature and humidity for business hours (8 am to 6 pm) local time. The full distribution of included data is illustrated in Fig. 2.

**Fig. 2:**
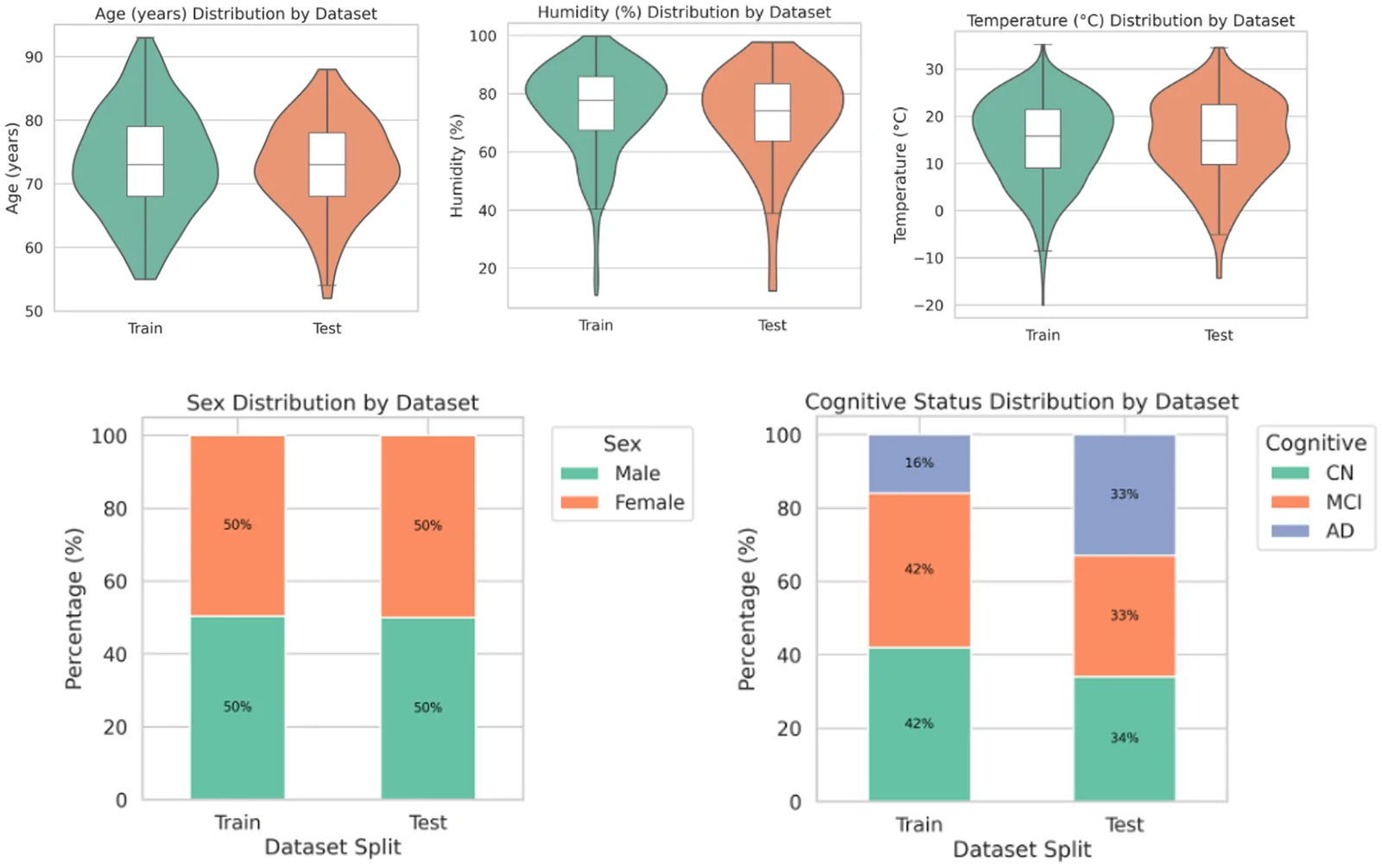
Distributions of the categorical variables sex and cognitive status, and violin plots of the distributions of continuous variables: age, temperature, and humidity. During the challenge, we released the age, sex, and cognitive status distributions for teams to consider when building their own datasets.

We acknowledge that data that teams may request access to typically consist of DICOM or NIFTI files, but we ask teams to use MHA images as inputs for their algorithms.

A limitation of the grand challenge as of October 2024 does not allow images to be uploaded as NIFTI, b-value, and b-vector files—instead, we upload a MHA file for the image and a shared .json file for the b-values and b-vectors for each image. We published conversion code to GitHub for participating teams and performed a quality assurance check in our private dataset to make sure that this formatting change did not corrupt data. For some approaches, the images may need to be converted back to NIFTI and bval/bvec formats for analysis, and we provided code for that conversion as well (https://github.com/MASILab/beyondFA_mlp). Before uploading to grand-challenge.org, all images were preprocessed with the PreQual pipeline^42^ and quality checked to avoid the accidental upload of a corrupted file from the NIFTI to MHA conversion. We recommended that participants preprocess their data with the PreQual pipeline before testing their algorithm to minimize the variation between their selected training data and the hidden test data on Ref. 43.

## 3 Testing Framework

Participants submitted Docker containers with their algorithm for final evaluation. Participant Dockers were expected to accept a MHA file containing a DW-MRI image, as well as .json files with relevant b-values and another with relevant b-vectors. Dockers should then output a json file with the name “features-128.json” that contains a single feature vector of 128 items (Fig. 3). If the selected feature required fewer than 128 features, we asked participants to zero-pad their outputs. No restrictions were placed on the feature extraction methodology, allowing submissions based on any diffusion metrics, tract-specific quantitative measures, dimensionality reduction techniques, or learned feature representations. Assessments were performed with a shallow MLP, which has been made publicly available to teams on GitHub to evaluate their algorithm on their own data if they wish. All submissions were evaluated with the same downstream evaluation pipeline.

**Fig. 3:**
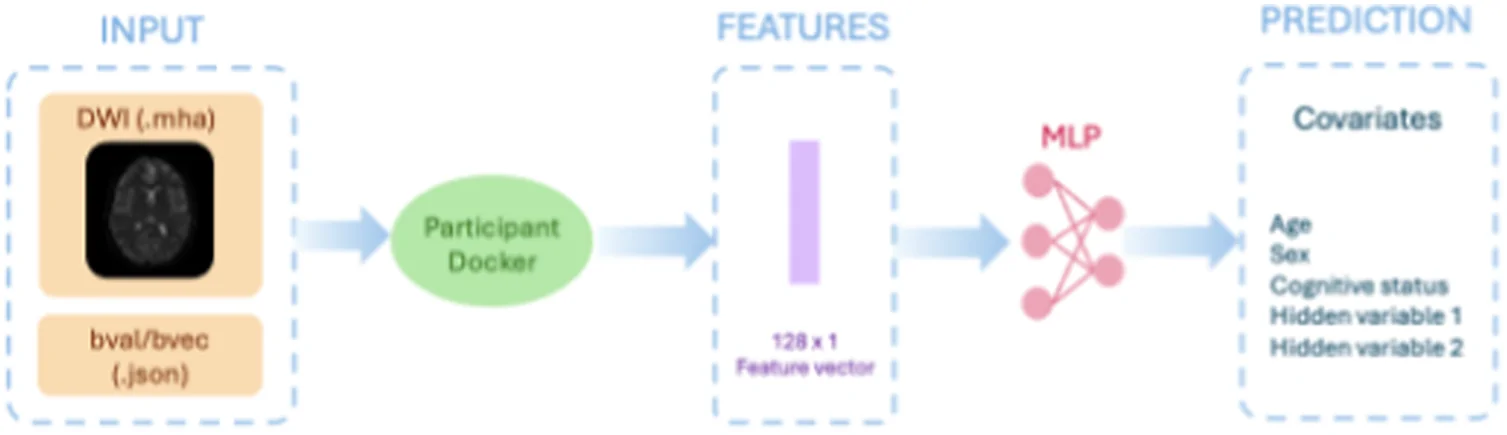
We provide an illustration of the overall workflow of the challenge. The end-to-end implementation, including the image archive, computational resources, and MLP output ranking, is hosted on grand-challenge.org/BeyondFA before we ran local evaluations. All steps except for the participant Docker were provided by the challenge organizers.

After processing the images, all the feature vectors were included in the evaluation implementation. We have predetermined a training set and a testing set from the secret data, so our algorithm assigns the same training and testing data for each submission. From there, we trained and tested a separate MLP classifier based entirely on the feature vectors from each algorithm submission and the associated age, sex, cognitive status, and secret variable labels (Fig. 3). The training included a deterministic setup with fixed random seeds to improve reproducibility. For each set of feature vectors, the script trained five separate MLPs (one per task). Each loss function was specific to the task assigned to each MLP: binary cross-entropy loss for sex, cross-entropy loss for cognitive status, and L1 loss for the three continuous variables (age, humidity, and temperature). Performance on downstream tasks was evaluated with task-appropriate metrics. Age, temperature, and humidity prediction were assessed using mean absolute error (MAE), which quantifies the average absolute difference between predicted and true values and is readily interpretable in the same units as the measurement. MAE was selected over mean squared error (MSE) because it retains this direct interpretability and does not disproportionately weight larger prediction errors. We evaluated sex and cognitive status prediction evaluated using standard classification accuracy, defined as the proportion of correctly classified samples. Standard accuracy was selected because it provides a direct and readily interpretable measure of overall classification performance and enables consistent comparison across participant submissions.

We provided a variety of baseline algorithms for teams to compare their results to. These algorithms have been published to the leaderboard on the grand-challenge.org webpage alongside the participants’ submissions as another comparison tool. We used raw scores to determine the highest-performing algorithm for age, sex, and cognitive status categories. In total, we provided two baseline algorithms and reported results for 12 additional submissions.

The Beyond FA Challenge was initially deployed on the Grand Challenge platform. Following the conclusion of the challenge period, the hosted leaderboard was no longer maintained. To ensure that all eligible submissions, including those received during conference outreach, were evaluated using an identical evaluation pipeline, the final rankings reported in this manuscript were generated through a local implementation of the challenge evaluation framework. Consequently, the results presented here may differ from the archived Grand Challenge leaderboard.

### 3.1 Baseline Algorithms

#### 3.1.1 Baseline Algorithm 1: Random Classification

Although we expect that teams will submit well-considered, domain-informed feature extraction methods for the AD data, we compare these methods to a random classification baseline as a sanity check. We submit a Docker that takes the training and testing data as input and generates random feature vectors without any informed domain decisions.

#### 3.1.2 Baseline Algorithm 2: FA Averaged across Regions of Interest

As our challenge is based on the need to consider AD feature representations beyond FA in an AD context, we use a standard FA computation as a second baseline. We understand that participants may use a region of interest (ROI)-based approach to prioritize brain regions that experience more significant changes during AD progression. As opposed to a standard baseline of a global brain FA map, we provided a more robust baseline. Here, we use TractSeg^26^ to generate white matter segmentations of 72 different brain regions and compute the average FA value for each of those brain regions. The FA computations are the rows in the output feature vector.

### 3.2 Submitted Algorithms

Each of the following method blocks was submitted and provided by challenge participants, who are the respective authors of each method (Secs. 3.3 to 3.13).

### 3.3 3D RotNet by Rotated Vine

This feature extraction method is based on a deep learning approach using a 3D convolutional neural network (CNN). We implemented an architecture inspired by RotNet,^44^ a self-supervised learning method, to learn robust feature representations directly from the FA images. The model was pre-trained to predict 3D rotations applied to the input volumes using diffusion MRI data from 55 subjects obtained from the Human Connectome Project.^4^^,^^45^ Using MRtrix3,^46^^,^^47^ we sequentially performed skull stripping, distortion and motion correction, and bias field correction to preprocess the diffusion MRI data, followed by tensor fitting and FA computation to obtain FA map. This self-supervised task allows the network to learn meaningful features relevant to 3D structure without relying on manual labels. The features learned during this pretraining phase were then used for evaluation.

### 3.4 SimSiam_Reprs by GSS_Greymatter

Because the challenge deals with optimal learning of image representations from the diffusion MRI data, we followed the Simple Siamese (SimSiam) approach as discussed by Chen and He.^48^ Simple Siamese architecture takes two randomly augmented views of an image, and the encoder network processes the two augmented views separately in two parallel branches. The encoder network combines a visual backbone and a projector MLP. The original paper discusses the SimSiam approach for 2D image data. We have modified their strategy to deal with 3D medical data for this challenge.

Because the diffusion MRI is a form of 3D data, we have used a 3D Vision Transformer as our visual backbone. The encoder network shares the weights between the two branches. A predictor MLP will process the output from the encoder. The Simple Siamese network minimizes the negative cosine similarity between each predictor output and the stop-gradient projection of the opposite branch, computed in both directions.

We trained the network for 100 epochs on a single NVIDIA L4 GPU. We used a batch size of 64. We used AdamW as our optimizer, with a weight decay of 1e−4 and momentum of 0.9. We used an initial learning rate of 0.0125, and it was decayed using a cosine scheduler.

### 3.5 Axial Diffusion by TheMix

We preprocessed the data by generating a brain mask, estimating the response function, and computing FODs. We segmented anatomically defined white matter bundles with TractSeg^26^ and computed average axial diffusion within each bundle.

### 3.6 TractoMD by SoloHenry

We used MRtrix3^46^^,^^47^ to compute a brain mask. Then, we estimated a single-tissue white matter response and reconstructed white matter FODs, then derived peak maps. Next, we ran TractSeg (pre-trained CNNs; no additional training by us)^26^ to obtain 72 bundle segmentations. We then fit a diffusion tensor and computed an MD volume with SCILPY.^49^ Using the TractSeg bundles, we computed the mean MD within each bundle, recorded zeros for empty masks, and collected all bundle-wise values. We sorted bundles in a fixed order, concatenated the values into a vector, and kept a uniform 128-feature interface by appending trailing zeros.

### 3.7 Tribundle by BlueMatter

We used MRtrix3^46^^,^^47^ to compute a brain mask, estimated a single-tissue white matter response function, and reconstructed white matter FODs before deriving peak maps. Then, we ran TractSeg^50^^–^^52^ out-of-the-box to obtain tract segmentations, start/end region masks, and tract orientation maps (TOMs), and performed bundle-specific tracking for six bundles (CG, UF, ILF; left/right). Next, we fit the diffusion tensor and computed FA/MD/AD/RD maps. Using TractSeg’s tractometry, we sampled each scalar along every bundle at N=5 equally spaced nodes between endpoints and aggregated across streamlines (median). We concatenated all values into a 120-D vector (6 bundles × 5 nodes × 4 metrics); to keep a uniform 128-D interface, we appended eight trailing zeros in a fixed order.

### 3.8 TensorCL-SE v1 and Tensor CLE-SE v2 by SoloHenry

We employed a contrastive learning framework with a 3D ResNet-18^53^ backbone enhanced by squeeze and excitation (SE) blocks. We generated two stochastically augmented 3D views of each diffusion tensor image as positive samples. We used simple but effective volumetric augmentations—blurring and brightness jitter—to expose the model to nuisance variation while preserving anatomy. We trained on 8000 DTI volumes and stopped after 8 epochs once the contrastive objective plateaus.

### 3.9 TractoPCA by SoloHenry

We used MRtrix3^46^^,^^47^ to compute a brain mask and estimate a single-tissue WM response function. After this, we fit the diffusion tensor and computed FA, MD, AD, and RD volumes with SCILPY.^49^ Next, we reconstructed white matter FODs, extracted peak orientations, and ran TractSeg^26^ to obtain 72 personalized white matter bundles. For every bundle–metric pair, we computed nine descriptive statistics—mean, median, std, skew, kurtosis, p10, p25, p75, and p90—and we concatenated all values into a 2592-dimensional vector. We then standardized this vector with a precalculated z-score scaler and projected it onto a precalculated PCA basis to 60 components. We kept a uniform 128-D interface by appending 68 trailing zeros in a fixed order.

### 3.10 Ensemble Average Propagator-Based Return To x Probabilities by EAP-RTxp

#### 3.10.1 Preprocessing

We computed fractional anisotropy (FA) using standard diffusion tensor imaging (DTI) fitted with the FSL dtifit tool (Analysis Group, FMRIB, Oxford, UK;^42^). The FA volumes were nonlinearly registered (fnirt),^54^,^55^ to the FMRIB58_FA template in the Montreal Neurological Institute (MNI) space, with an initial linear registration (flirt)^56^ under seven degrees of freedom, using a normalized correlation cost function and spline interpolation. We then computed a proxy for the inverse mapping and used it to warp the Johns Hopkins University (JHU) white matter atlas labels^57^ to each subject’s native space.

#### 3.10.2 Feature extraction

For each subject, three groups of ensemble average-based propagator characteristics were extracted, namely, the full, axial, and planar moments. All measures were computed from single-shell diffusion-weighted MRI data at $b=1000\;s/{\mathrm{mm}}^{2}$ using the generalized AMURA framework.^58^ The generalized AMURA approach defines microstructural measures under the assumption that diffusion anisotropy does not depend on the b-value.

We computed the following moments of p-th order:

Full moments: p=0 (RTOP; Return-To-the-Origin Probability), p=1/2, p=1, and p=2,

Axial moments: p=0 (RTPP; Return-To-Plane Probability), and p=1,

Planar moments: p=0 (RTAP; Return-To-Plane Probability), and p=1.

To form the feature vector for each subject, we computed the median values of the eight measures across 16 carefully selected JHU white matter regions that have been reported in previous studies to show statistically significant differences between Alzheimer’s disease cases and healthy controls. These regions include the genu, body, and splenium of the corpus callosum, as well as thirteen regions from both hemispheres: the anterior and posterior limbs of the internal capsule, the retrolenticular part of the internal capsule, the anterior, superior, and posterior corona radiata, the sagittal stratum, the external capsule, the cingulum (cingulate gyrus), the cingulum (hippocampus), the superior longitudinal fasciculus, the uncinate fasciculus, and the tapetum.

### 3.11 NODDI-SMT-UNet by Al-zheimer

#### 3.11.1 Preprocessing

The ICBM-DTI-81 white-matter atlas^44^ was registered into the subjects’ native space. The diffusion-weighted MRI data were initially masked (FSL, bet^57^), followed by the calculation of fractional anisotropy (FSL and dtifit). The FA was then registered to standard space nonlinearly preceded by a linear fit (FSL, flirt, and fnirt^54^^,^^55^). Inversed non-linear mapping was applied to register the atlas to the patients’ native space. Next, the masked diffusion data were interpolated using spherical harmonics. The order of the spherical harmonics decomposition was set to 6, and a regularization parameter of 0.006 was applied to the Laplace–Beltrami regularization. In addition, a template was utilized that contained 32 gradient directions for a single b-value, generated using the Q-Space Sampling tool.^59^

#### 3.11.2 Data analysis

The NODDI-SMT-UNet was utilized to estimate the parameters of the NODDI^60^ and SMT^61^ models. Specifically, we estimated the orientation dispersion index, isotropic, intra-neurite, and extra-neurite volume fractions of the NODDI model, and intra-axonal volume fraction of the SMT model. Finally, for each microstructural parameter, the mean value within selected regions of interest from the atlas, which were previously identified as statistically significant regions based on TBSS analysis,^62^ was calculated and assembled into a vector consisting of 128 values.

The NODDI-SMT-UNet model is based on the standard UNet architecture. It takes normalized diffusion data (divided by b0) as input, which includes 32 gradient directions at a b-value of $1000\;s/{\mathrm{mm}}^{2}$. The model outputs five microstructural parameters derived from the NODDI and SMT models. The encoder begins with an initial block of 64 channels, and the number of channels doubles consistently until it reaches 512 channels at the bottleneck. The decoder mirrors this structure. This model was trained using data from 30 subjects from the ADNI^7^ dataset (10 CN, 10 MCI, and 10 AD). The model was then tested on data from another 23 subjects from the same dataset (8 CN, 8 MCI, and 7 AD).

### 3.12 Free Water Elimination by Ofer Pasternak, by Damp No More

The initial feature extraction involved calculating the free water fraction (FWF) across the dataset. This was performed using an implementation of the free water elimination (FWE) model proposed by Paskernak et al (2009).^63^ The method models the diffusion signal as a combination of two compartments: tissue and an isotropic free water component, allowing the estimation and subtraction of the free water signal to obtain corrected tissue diffusion metrics.

### 3.13 FWF-BundleParc-NF-Neuro by SCIL Team 6

Inputs were converted to gradient tables and NIFTI images. The diffusion volume was spatially resampled to 2 mm isotropic. b0 images were extracted and we ran FSL BET^57^ to obtain a brain-extracted diffusion volume and brain mask. Free-water correction (from AMICO^64^^,^^65^ toolbox) was computed, and DTI maps were computed using SCILPY^49^ from the free-water-corrected diffusion volume. This provides FWF, FA, and MD maps. Transformations (rigid, affine, and diffeomorphic) from MNI to subject space were computed, using the b0 and FA volumes (Mutual Information) as references. The transformation is then used to register the BundleParc atlas in MNI to the subject space. Finally, the average FWF is extracted in up to 128 regions of the BundleParc atlas in the subject space, i.e., four parcels for each of 32 white-matter bundles.

We have included Table 1 as a summary of submitted algorithm approaches. Some submissions used PreQual as the recommended preprocessing method, while others elected to use their own preprocessing, as indicated in the table. The challenge submissions encompassed several broad methodological strategies (Table 1), ranging from conventional diffusion-derived features to microstructural modeling and self-supervised deep learning representations. A substantial subset of methods summarized diffusion measures within white matter bundles, differing primarily in the diffusion measures used and subsequent feature processing. Other submissions incorporated more explicit microstructural modeling, including free-water and NODDI/SMT-derived representations. In contrast, the deep learning approaches used CNN-, transformer-, or contrastive-learning-based strategies to derive representations directly from the imaging data. Thus, the submissions differed not only in their specific algorithms but also in the level at which information was represented, from predefined diffusion and microstructural measures to learned image representations. Most submissions used PreQual preprocessing, although several methods operated without it, providing additional methodological heterogeneity across challenge entries.

**Table 1:** Summary of submissions.

Method	Primary algorithm type	Used PreQual?
Random Inputs Baseline	Random baseline (no learned features)	×
FA baseline	Bundle-wise diffusion metric	✓
3D RotNet	Deep learning (CNN)	✓
SimSiam_Reprs	Deep learning (vision transformer)	✓
Axial diffusion	Bundle-wise diffusion metric	✓
TractoMD	Bundle-wise diffusion metric	✓
Tribundle	Bundle-wise diffusion metric	✓
TensorCLE-SE v1	Deep learning (contrastive)	✓
TensorCLE-SE v2	Deep learning (contrastive)	✓
TractoPCA	Bundle-wise diffusion metrics + PCA	✓
EAP-RTxp	Bundle-wise diffusion metric	×
NODDI-SMT-UNet	Deep learning microstructure estimate + bundle features	×
Free Water Elimination	Microstructure estimation	✓
FWF-BundleParc-NF-Neuro	Free water correction + bundle-wise diffusion metrics	×

## 4 Standings for Each Variable

We show the standings in Table 2 and plot the standings with ChallengeR software^66^ with full plots available in Supplementary Material A–E.

**Table 2:** First, second, and third place for each task.

Variable	First place	Second place	Third place
Age	NODDI-SMT-UNet	EAP-RTxp	FWF-BundleParc-NF-Neuro
Sex	TensorCLE-SE v1	FWF-BundleParc-NF-Neuro	FA baseline
Cognitive status	TensorCLE-SE v1	TensorCLE-SE v2	EAP-RTxp
Temperature	NODDI-SMT-UNet	SimSiam_Reprs	FA baseline
Humidity	EAP-RTxp	SimSiam_Reprs	Random inputs baseline

TractoPCA and free water elimination were excluded from summary analyses and comparative visualizations of the regression tasks because their prediction errors were extreme outliers, several orders of magnitude larger than those of the other submissions (e.g., age MAE>29,000 years and temperature MAE>66,000 units). Including these values on the same scale would obscure differences among the remaining methods. We mention these results for completeness but omit them from comparative visualizations because they would dominate the scale and obscure differences among the remaining methods. The extreme errors likely reflect characteristics of the submitted feature representations rather than meaningful predictive performance. In particular, differences in feature scaling, feature distributions, or the presence of extreme feature values may have resulted in unstable regression predictions when the standardized downstream MLP evaluation was applied. Because the same evaluation pipeline and fixed training and testing sets were used for all submissions, these results may indicate that these feature representations were not well suited to the common downstream regression framework without additional submission-specific preprocessing or normalization.

## 5 Biomarker Correlation

We did not tell teams that we would search for their top correlated features to known AD pathology, but we anticipated that their classification-driven algorithms could correlate with amyloid beta 42, ptau, and tau CSF readings. We show the distributions for these variables in our dataset in Fig. 4 with 46 measurements for ABETA and 47 measurements for TAU and PTAU. Notably, the distributions presented show consistency with Alzheimer’s disease literature: ABETA42 decreases as TAU and PTAU increase.^67^^,^^68^

**Fig. 4:**
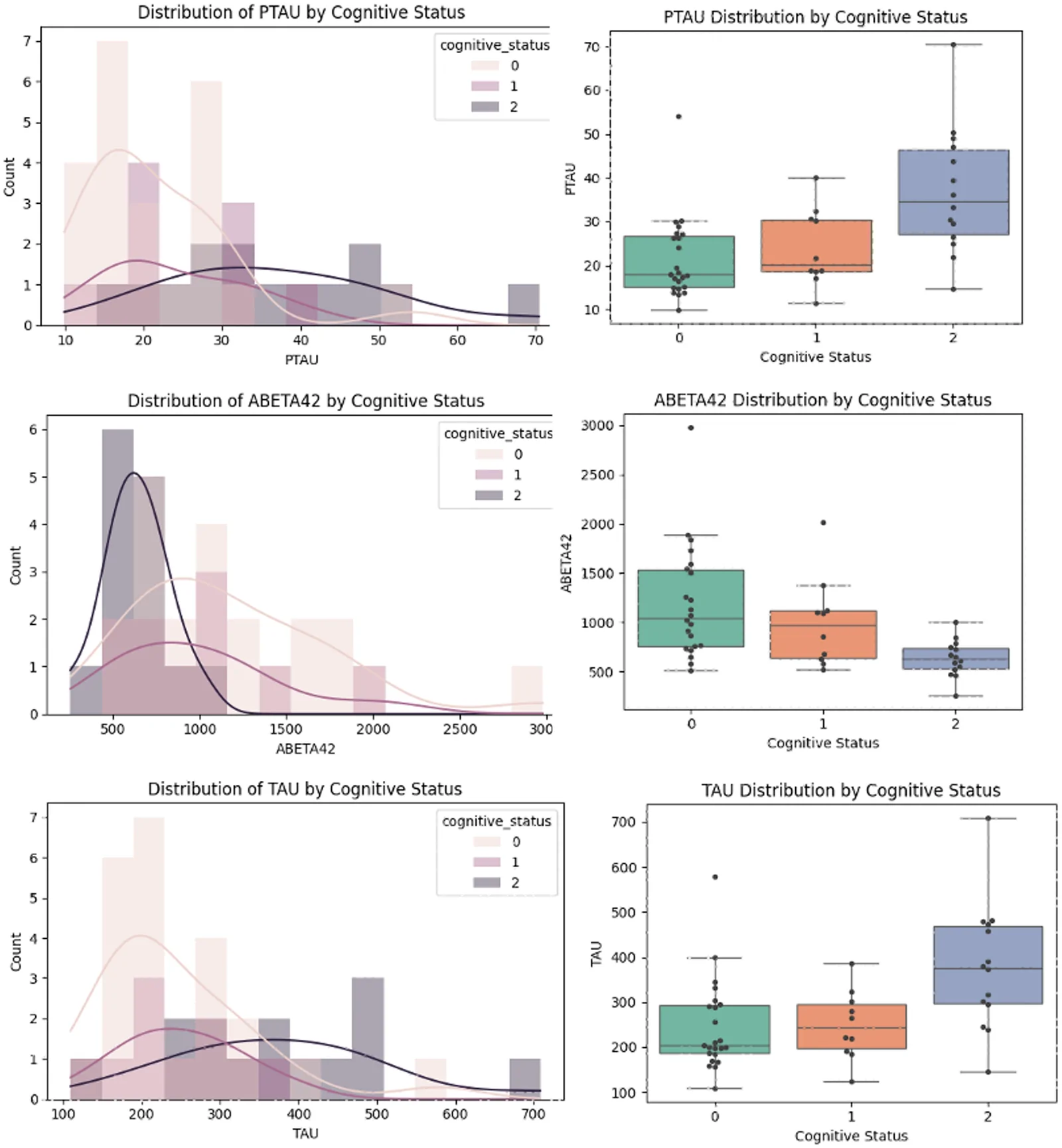
Distributions of subjects with cerebrospinal fluid (CSF) biomarkers amyloid beta 42, tau, and ptau across cognitive status groups: cognitively normal (0), mild cognitive impairment (1), and Alzheimer’s disease (2) subjects. The left panel shows a histogram of the dataset by study group, whereas the right panel shows boxplots summarizing the median, interquartile range, and distribution measurements within each group. The relationship between cognitive status and amyloid beta 42, tau, and ptau within the challenge dataset reflects those expected from AD literature.

We computed Pearson and Spearman correlation coefficients for each feature submitted to the challenge and present the results for only the algorithms with at least one statistically significant correlation with any of the CSF metrics (amyloid beta 42, tau, and ptau) following false discovery rate (FDR) correction. We highlight algorithms with at least one statistically significant correlation following FDR correction to highlight feature representations demonstrating detectable associations with the CSF biomarkers. Algorithms without significant correlations should not necessarily be interpreted as lacking biologically relevant information; rather, their submitted features did not demonstrate statistically significant univariate associations with the CSF measures under the present analysis. These representations may encode information that is distributed across multiple features, nonlinear, or otherwise not captured by individual Pearson or Spearman correlations. We find seven positive results (q<0.05) for TensorCLE-SE v1 for ABETA42 Spearman correlation (Supplementary Material F) and two positive results for Pearson correlation (Supplementary Material G). FWF-BundleParc-NF-Neuro had seven positive results for TAU Spearman correlation (Supplementary Material H), one positive result for TAU Pearson correlation (Supplementary Material I), and one positive result for PTAU correlation coefficient (Supplementary Material J). TractoPCA resulted in one positive result for ABETA42 Spearman correlation (Supplementary Material K). We report Pearson and Spearman correlation for all algorithms in Supplementary Material L.

## 6 Discussion

Although no approach won overall, several approaches showed increased sensitivity to the biomarkers of interest. Moving beyond FA requires task-dependent consideration of appropriate metrics. Although we expected age and cognitive status prediction performances to show high alignment, only one algorithm (EAP-RTxP) is ranked in the top three between the two categories. The accuracy of the tri-classification cognitive status task for the algorithms in this work is substantially lower than the accuracy presented in binary classification settings,^12^^,^^35^^,^^69^ however, our tri-classification results align with performances within AI4D,^32^ emphasizing the importance of approaching these problems with domain knowledge and nuance to move forward with tri-classification.

Even though similar tri-classification results are reported, there are several findings unique to this challenge compared with AI4AD. AI4AD’s submission requested the development of a classification method for a predetermined quantitative bundle of metrics, but the top two cognitive status accuracy methods in this work are whole-image contrastive learning methods, whereas the third is a bundle-based metric not used in AI4AD. Notably, AI4AD took place within a year of the paper that launched contrastive learning for computer vision,^58^ emphasizing that the neuroimaging community should revisit AD classification methods as new approaches are introduced. With further refinement after a first attempt in the Beyond FA Challenge, these methods may show promise for future cognitive status classification tasks.

All three of the top methods for age used bundle-wise metrics more complex than the standard DTI-derived FA, drawing inspiration from estimation of NODDI parameters, a full water molecule displacement profile estimation, and free water fraction. Although past approaches have relied on FA due to limited data quality, methods have evolved to include deep learning estimators of methods such as NODDI that would have required multi-shell or high angular resolution data. In age estimation, we learn that bundle-wise resolution of these metrics (as performed in the FA baseline) is appropriate; the mean absolute error (MAE) from the FA baseline aligns with performance from previous work.^70^

Sex, humidity, and temperature prediction top algorithms span whole-image contrastive learning approaches and bundle-wise metric computations, but the random inputs baseline shows more correlation with humidity than many of the submissions with top predictions for other variables. The relatively high MAE for humidity suggests that the features submitted to the challenge do not correlate with humidity, but nine out of 12 submissions have an MAE of less than 10 degrees Celsius for the temperature variable. We learn that temperature and these feature representations are not entirely unrelated, and encourage future study in the relationships between the environmental conditions and DW-MRI.

We also found that the correlation between the features submitted to the challenge has statistically significant relationships with amyloid beta 42, tau, and ptau. Although TractoPCA’s features failed to be useful in a continuous prediction task, its features are still useful for a correlation task. Although no single feature extraction method yielded correlation with all three biomarkers, all three were represented. Two of the three submissions with correlations with these unknown variables use bundle-wise metric computation, whereas the third is a contrastive learning method, highlighting again why challenges like this matter: when we combine relatively new computer vision methods with an AD focus, what can we learn about AD feature representations?

Taken together, the Beyond FA Challenge provides several insights into how DW-MRI feature representations should be evaluated and interpreted. Rather than identifying a single representation that is uniformly optimal across applications, the challenge highlights substantial task dependence in the utility of different representations. The results further reveal differences in the performance of methodological families across downstream targets, distinctions between predictive performance and feature-level biological associations, and relatively small absolute differences among many highly ranked methods. We discuss these findings below in terms of four broader themes.

### 6.1 Representation Utility Is Dependent on the Downstream Task

No submission dominated across all evaluation tasks, and the relative performance of methods varied across demographic, cognitive, environmental, and biomarker-related outcomes. Moreover, the relatively small performance differences among many highly ranked methods suggest that increased methodological complexity did not consistently translate into substantially greater downstream predictive information. These findings support evaluating DW-MRI representations across multiple complementary tasks rather than assuming that performance on a single benchmark reflects their overall utility.

### 6.2 Optimal Representation Strategy May Depend on the Information Targeted by the Downstream Task

The relative performance of methodological families varied by downstream target. Bundle-wise representations were particularly successful for age prediction, with all three highest-ranked methods incorporating diffusion or microstructural information summarized within white matter bundles. In contrast, the two highest-performing approaches for cognitive status used whole-image contrastive learning, suggesting that spatially distributed information not captured by predefined tract summaries may be informative for disease-related classification. The remaining tasks showed less consistent separation between methodological families, with both learned whole-image and engineered bundle-wise representations appearing among the stronger-performing approaches. Collectively, these results suggest that the optimal spatial scale and representation strategy may depend on the biological or technical information targeted by the downstream task.

### 6.3 Predictive Performance and Biological Association Capture Complementary Properties of a Representation

The discrepancy between downstream prediction performance and feature-level biomarker correlations further demonstrates that representation quality is not necessarily captured by a single evaluation paradigm. For example, although TractoPCA produced unstable predictions for the continuous regression tasks, individual TractoPCA features nevertheless demonstrated significant associations with CSF biomarkers. Thus, poor performance when a representation is coupled to a particular downstream model does not necessarily indicate an absence of biologically relevant information. Conversely, strong predictive performance does not establish that individual features have direct biomarker associations. Evaluating representations using complementary predictive and association-based analyses may therefore provide a more complete characterization of the information they encode.

### 6.4 Rank Alone May Overstate Practical Differences between Representations

Although the challenge produced an ordinal ranking for each task, the absolute differences among several highly ranked submissions were small. Accordingly, rank position alone may overstate practical differences in representation quality. This is particularly relevant given that methods with substantially different computational strategies achieved similar downstream performance. Rather than identifying a single superior representation, the challenge suggests that several current approaches capture overlapping information relevant to these downstream targets.

### 6.5 Overall Implications

Collectively, these findings suggest that the value of a DW-MRI representation cannot be reduced to a single ranking, downstream task, or evaluation criterion. Representation choice should instead be considered in relation to the intended application, the spatial and biological information of interest, and whether the goal is prediction, biological interpretation, or both. More broadly, the Beyond FA Challenge provides a framework for evaluating representation methods across complementary tasks and evaluation paradigms, while also highlighting areas in which future benchmarks could further distinguish between approaches.

## 7 Limitations

We used ADNI for our evaluation and testing because this initiative includes enough data to support our effort.^8^ Although the identity of the evaluation dataset was not disclosed to participants, the use of ADNI as the hidden test set introduced a source of bias favoring participants who developed or optimized their methods using ADNI data. The dataset identity was withheld to encourage approaches that generalize across AD cohorts rather than tailored methods for ADNI and constraints due to data use agreements. While the majority of submitted methods relied on handcrafted diffusion MRI features rather than large-scale learned representations, potentially reducing opportunities for dataset-specific optimization, this possibility cannot be excluded and should be considered when interpreting the challenge rankings. Future challenges would benefit from an evaluation on an independent cohort that is inaccessible to all participants to further reduce this source of bias.

In addition, this challenge introduces several avenues for further investigation of environmental factors. Future work assessing the utility of diverse DW-MRI-derived features and their relationships with environmental variables across more datasets could establish generalizability of the findings presented to this study. We imbalanced the training/testing examples due to real-world data collection limitations. Some of the feature extraction methods struggled to produce reasonable estimates within the continuous variables in our dataset, and future MLP-based challenges should control for variance in feature ranges, especially because these algorithms produced reasonable predictions for the classification variables. We generalized the weather information across an entire metropolitan area for the business hours of a single day, so further study of sensitivity of DW-MRI variables to weather is needed as well as a thorough evaluation of how these variables confound with individual site.

Although the downstream evaluation tasks were selected because they reflect biologically relevant demographic, disease, and environmental variables, the challenge was designed to benchmark computational feature representations rather than evaluate clinical decision support. Consequently, the reported performance metrics should be interpreted as measures of representation quality and not as indicators of clinical utility. Future studies should incorporate clinician input to assess the practical relevance, interpretability, and potential clinical integration of these representations. An important observation from this challenge is the relatively small performance differences among the highest-ranked methods. Despite employing substantially different feature extraction strategies, many approaches captured similar information relevant to the downstream prediction tasks. These findings indicate that future benchmark datasets or other downstream tasks may be necessary to better distinguish among state-of-the-art MRI representations.

We cannot release the data for open access, though we can provide lists of the training and testing data to those with appropriate permissions to the data involved. We understand that the lack of a single dataset for all participants may be a limitation of our approach, but we aimed to include as many researchers across biomedical imaging and machine learning as possible. These diverse groups may have limited access to different datasets, and we did not wish for our challenge to be limited only to those with certain data permissions.

## 8 Conclusion

We evaluated 14 algorithms submitted from the USA, France, Poland, Spain, India, and Canada. This international effort identified imaging feature representations for AD that outperform FA in age and sex prediction tasks and others that correlate to known AD biomarkers. We presented a method to evaluate the challenge results in cases with limited or private data, and we evaluated correlation of DW-MRI measures with environmental variables for the first time. Notably, three bundle-wise metric extraction methods outperformed the similarly designed FA baseline method in the age task. This challenge facilitated the unique opportunity to directly compare quantitative imaging metrics and novel deep learning approaches, and the results have shown an exciting new avenue for whole-brain contrastive learning to predict cognitive status in a tri-classification task. We thank each team for their creative and meaningful contribution to this work, and we encourage the imaging community to think beyond FA for meaningful imaging feature representations for AD pathology.

## Supplementary Material

10.1117/1.JMI.13.5.054003.s01

## Data Availability

ADNI data requires permission from its governing bodies. MASiVAR data is available on OpenNeuro (https://openneuro.org/datasets/ds003416/). Evaluation code available to teams can be accessed via GitHub (https://github.com/MASILab/BeyondFA_eval). The official challenge home page is https://bfa.grand-challenge.org/.
