# Supplementary material for: Contrastive learning and advanced bundle metrics outperform fractional anisotropy for Alzheimer’s disease diffusion MRI prediction: findings from the Beyond FA challenge
Source: J Med Imaging (Bellingham). 2026 Oct 15;13(5):054003. doi: 10.1117/1.JMI.13.5.054003 (PMC13671859; doi:10.1117/1.JMI.13.5.054003)
Supplement: Supplementary file 1 [file JMI_013_054003_SD001.docx]

Supplemental Material

Supplemental Material A: Age leaderboard.

Supplemental Material B: Sex leaderboard.

Supplemental Material C: Cognitive status leaderboard.

Supplemental Material D: Temperature leaderboard.

Supplemental Material E: Humidity leaderboard.

Supplemental Material F: Seven positive results for **TensorCLE-SE v1** for ABETA42 Spearman correlation.

Supplemental material G: Figure 7.1.2: Two positive results for **TensorCLE-SE v1** for ABETA42 Pearson correlation.

Supplemental material H: **FWF-BundleParc-NF-Neuro** had seven positive results for PTAU Pearson correlation for feature 1.

f22 (ranks)

Supplemental material I: **FWF-BundleParc-NF-Neuro** had seven positive results for TAU Spearman correlation.

Supplemental material J: **FWF-BundleParc-NF-Neuro** had one positive result for TAU Pearson correlation.

Supplemental material K: **TractoPCA** had one positive result for ABETA42 Spearman correlation.

| Method | Biomarker | Top Pearson Feature | Pearson rho | Top Spearman Feature | Spearman rho |
| --- | --- | --- | --- | --- | --- |
| SimSiam_Reprs | ABETA42 | f127 | 0.19 | f127 | 0.23 |
| Random Input Baseline | ABETA42 | f106 | 0.40 | f40 | 0.38 |
| FA baseline | ABETA42 | f16 | -0.22 | f62 | -0.24 |
| 3D RotNet | ABETA42 | f71 | 0.07 | f71 | 0.08 |
| Axial diffusion | ABETA42 | f49 | 0.28 | f42 | 0.34 |
| FWF-BundleParc-NF-Neuro | ABETA42 | f43 | -0.39 | f43 | -0.37 |
| Tribundle | ABETA42 | f18 | 0.29 | f18 | 0.38 |
| TractoMD | ABETA42 | f29 | 0.27 | f51 | 0.39 |
| TractoPCA | ABETA42 | f19 | 0.29 | f50 | 0.49 |
| TensorCLE-SE v1 | ABETA42 | f115 | -0.49 | f20 | 0.54 |
| TensorCLE-SE v2 | ABETA42 | f31 | -0.35 | f78 | -0.43 |
| EAP-RTxp | ABETA42 | f55 | 0.39 | f101 | 0.42 |
| NODDI-SMT-UNet | ABETA42 | f67 | 0.34 | f67 | 0.42 |
| Method | Biomarker | Top Pearson Feature | Pearson rho | Top Spearman Feature | Spearman rho |
| SimSiam_Reprs | TAU | f127 | -0.29 | f127 | -0.32 |
| Random Input Baseline | TAU | f39 | -0.34 | f59 | -0.37 |
| FA baseline | TAU | f43 | -0.25 | f43 | -0.32 |
| 3D RotNet | TAU | f71 | -0.16 | f106 | -0.13 |
| Axial diffusion | TAU | f34 | 0.27 | f22 | -0.29 |
| FWF-BundleParc-NF-Neuro | TAU | f1 | 0.50 | f17 | 0.46 |
| Tribundle | TAU | f6 | -0.28 | f18 | -0.37 |
| TractoMD | TAU | f34 | 0.27 | f22 | -0.29 |
| TractoPCA | TAU | f30 | -0.35 | f37 | 0.38 |
| TensorCLE-SE v1 | TAU | f87 | 0.34 | f101 | 0.38 |
| TensorCLE-SE v2 | TAU | f92 | -0.42 | f9 | 0.34 |
| EAP-RTxp | TAU | f117 | -0.31 | f117 | -0.37 |
| NODDI-SMT-UNet | TAU | f18 | -0.30 | f63 | -0.39 |

| Method | Biomarker | Top Pearson Feature | Pearson rho | Top Spearman Feature | Spearman rho |
| --- | --- | --- | --- | --- | --- |
| SimSiam_Reprs | PTAU | f127 | -0.32 | f106 | 0.35 |
| Random Input Baseline | PTAU | f117 | 0.35 | f59 | -0.35 |
| FA baseline | PTAU | f43 | -0.26 | f43 | -0.26 |
| 3D RotNet | PTAU | f71 | -0.19 | f106 | -0.16 |
| Axial diffusion | PTAU | f34 | 0.31 | f22 | -0.26 |
| FWF-BundleParc-NF-Neuro | PTAU | f1 | 0.50 | f17 | 0.44 |
| Tribundle | PTAU | f6 | -0.29 | f18 | -0.38 |
| TractoMD | PTAU | f34 | 0.31 | f22 | -0.28 |
| TractoPCA | PTAU | f30 | -0.37 | f37 | 0.45 |
| TensorCLE-SE v1 | PTAU | f87 | 0.34 | f101 | 0.41 |
| TensorCLE-SE v2 | PTAU | f92 | -0.38 | f9 | 0.30 |
| EAP-RTxp | PTAU | f117 | -0.33 | f117 | -0.41 |
| NODDI-SMT-UNet | PTAU | f18 | -0.30 | f63 | -0.37 |

Supplemental material L: Summary of the features most correlated with the intended biomarkers from each submission.
